# Supplementary material for: Photo-induced enhancement of lattice fluctuations in metal-halide perovskites
Source: Nat Commun. 2022 Feb 23;13:1019. doi: 10.1038/s41467-022-28532-0 (PMC8866428; doi:10.1038/s41467-022-28532-0)
Supplement: Supplementary file 1 — Supplementary Information [file 41467_2022_28532_MOESM1_ESM.pdf]

Supplementary Information

Photo-induced enhancement of lattice fluctuations in metal-halide perovskites

Mingcong Wang,[1,*] Yajun Gao,[1] Kai Wang,[1] Jiang Liu,[1] Stefaan De Wolf,[1] Frédéric Laquai[1,*]

[1]King Abdullah University of Science and Technology (KAUST), KAUST Solar Center (KSC), Physical Sciences and Engineering Division (PSE), Material Science and Engineering Program (MSE), Thuwal 23955-6900, Kingdom of Saudi Arabia

Corresponding authors: mingcong.wang@kaust.edu.sa; frederic.laquai@kaust.edu.sa

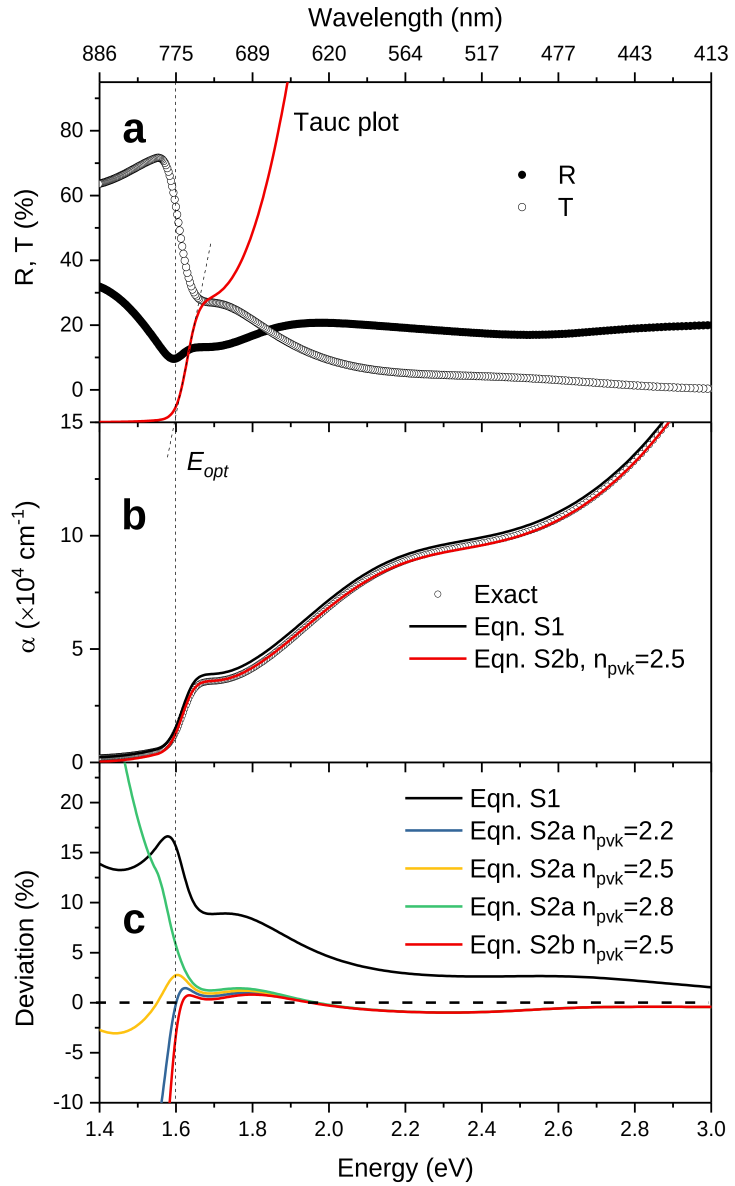

Supplementary Figure 1: (**a**) Reflectance (R) and transmittance (T) simulated with the reported complex refraction index of $MAPbI_3$ thin films using Eqn. S4. The red line is a Tauc plot that defines an optical bandgap (E_{opt}) of ~1.6 eV. (**b**) Calculated absorption coefficient using Eqn. S1 (black line) and S2b with a constant refractive index of n_{pvk}=2.5 (red line), respectively. (**c**): Comparison of deviation indicating that Eqn. S2a is more accurate than Eqn. S1 (black line). The deviation of Eqn. S2a depends on the value of n_{pvk}, however, it is not sensitive to it. For energies above E_{opt}, Eqn. S2a can be approximated as Eqn. S2b (red line).

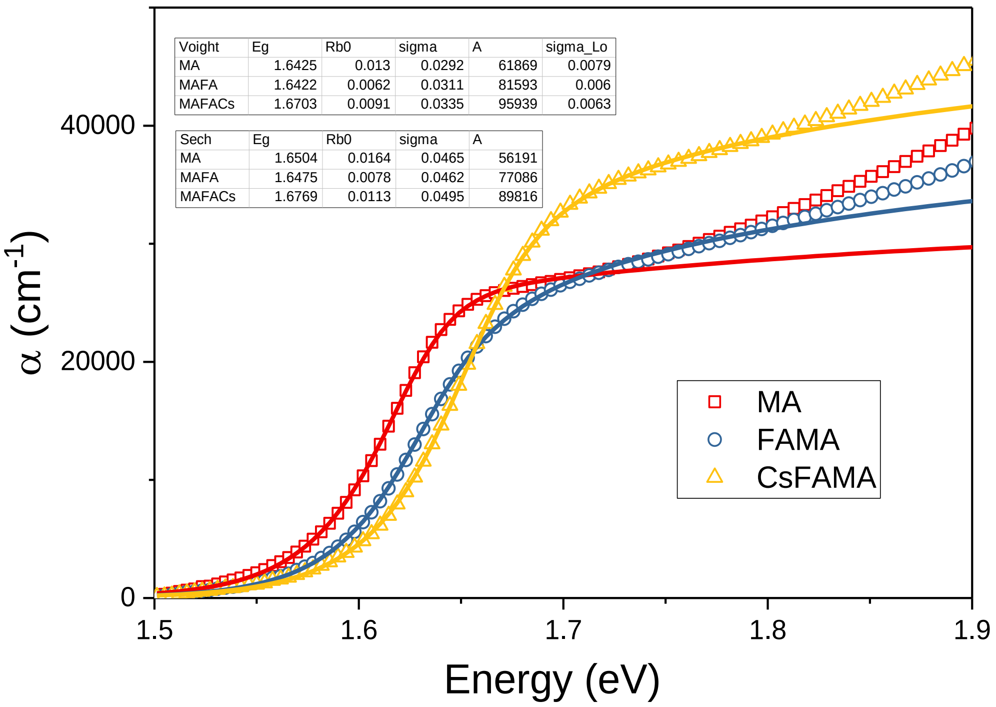

Supplementary Figure 2: Elliott fits to the ground-state absorption spectra. The inset tables list the fit results obtained using two different broadening functions: the Voight distribution and the hyperbolic-secant distribution.

Film thickness: ~300 nm

MA: $MAPbI_3$

FAMA: $(FA_{0.83}MA_{0.17})Pb(I_{0.83}B\ r_{0.17})_3$

CsFAMA: $(Cs_{0.05}FA_{0.8}MA_{0.15})Pb(I_{0.85}Br_{0.15})_3$

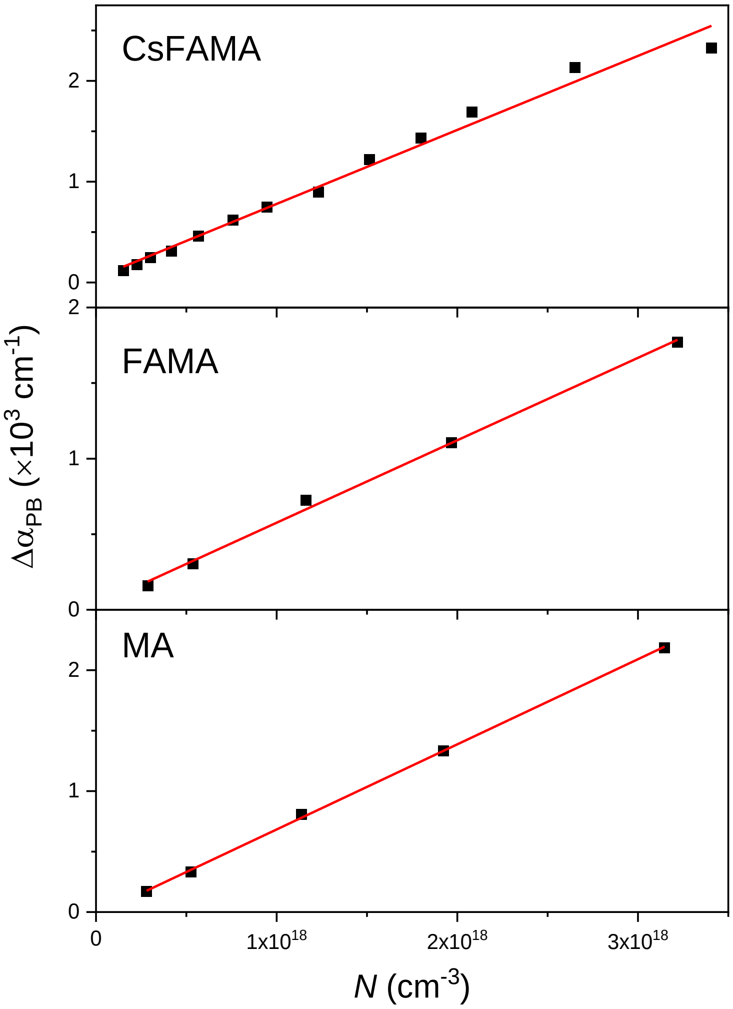

Supplementary Figure 3: The maximum photobleach of ground-state absorption ($\Delta\alpha_{PB}$) increases linearly with carrier density (N), indicating our experiments are free of high-fluence saturation.

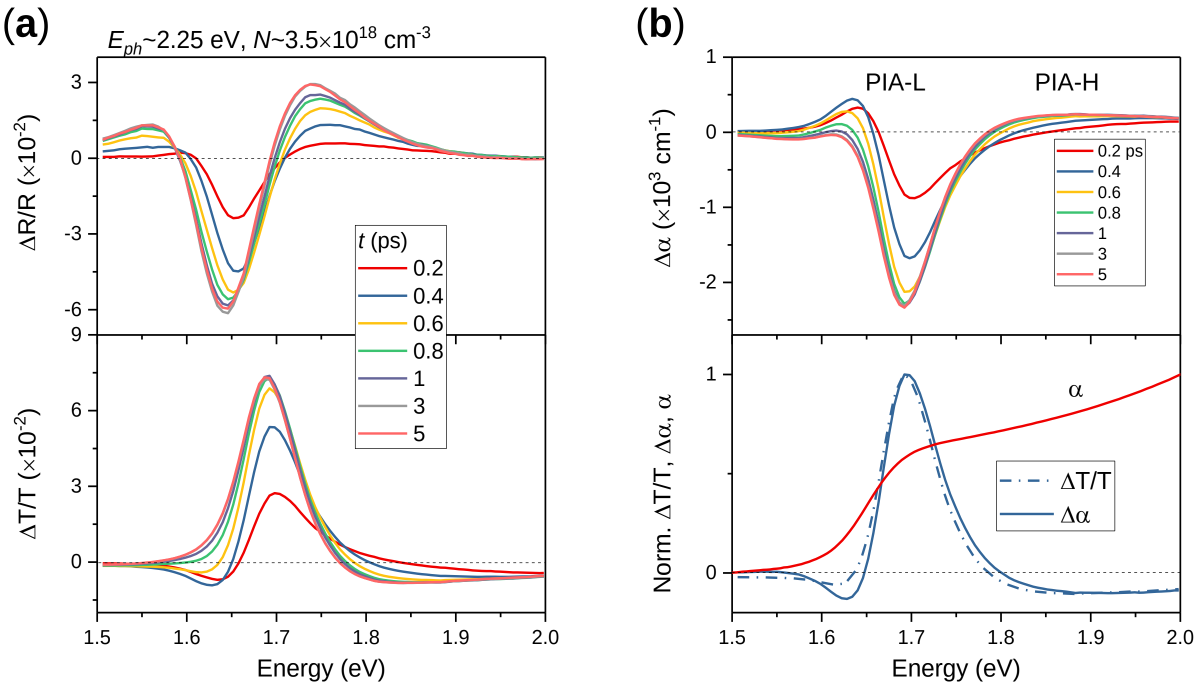

Supplementary Figure 4: Transient absorption spectra of CsFAMA thin films (thickness ~300 nm). (**a**) Measured transient reflectivity (ΔR/R) and transient transmissivity (ΔT/T) at different time delays. (**b**) Transient absorption spectra (Δα) extracted from ΔR/R and ΔT/T using Eqn. S12. Bottom panel: comparison between ΔT/T and Δα at t=0.4 ps. Red solid line: the normalized spectrum of the ground-state absorption coefficient.

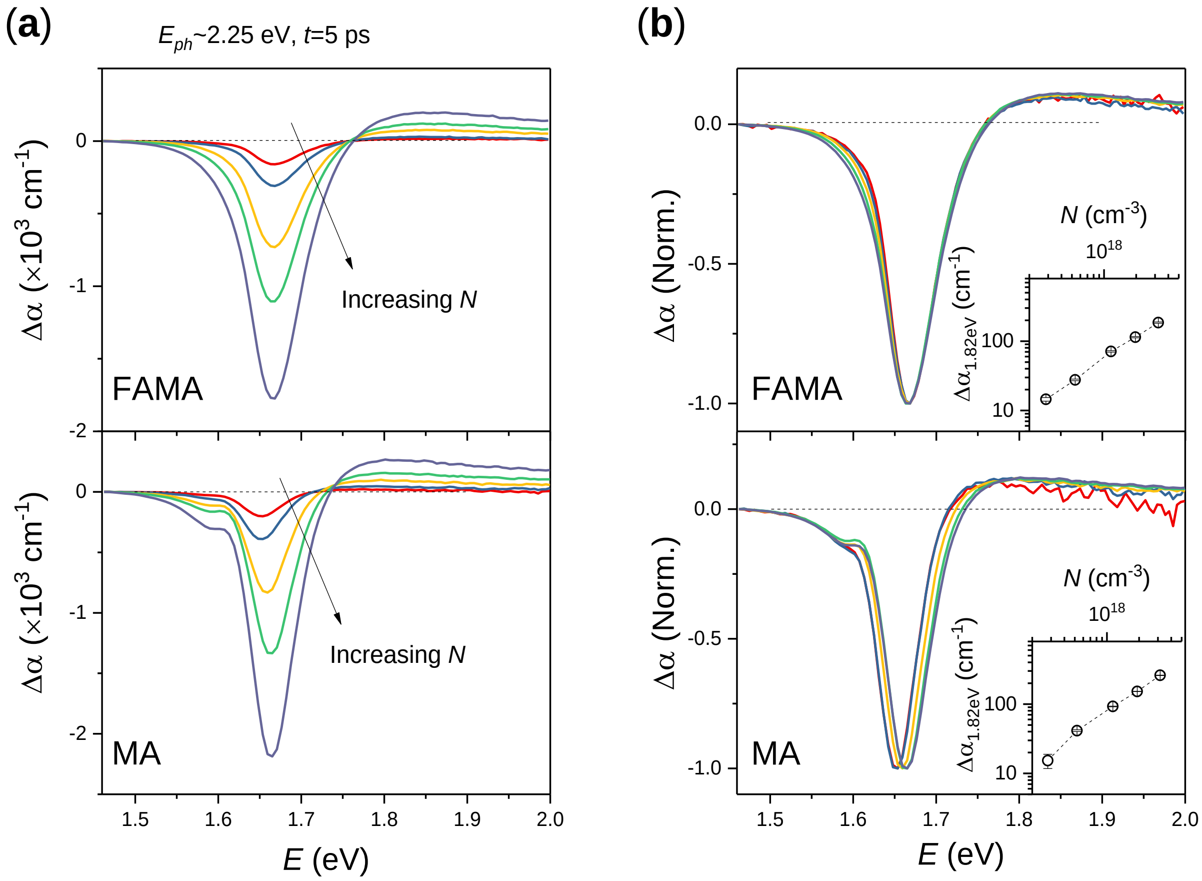

Supplementary Figure 5: (a) Transient absorption spectra of FAMA and MA thin films, respectively. (**b**) The normalized transient absorption spectra show that the photo-induced absorption (PIA) above ~1.8 eV scales with carrier density. Insets: the PIA amplitude at E=1.82 eV linearly increases with carrier density.

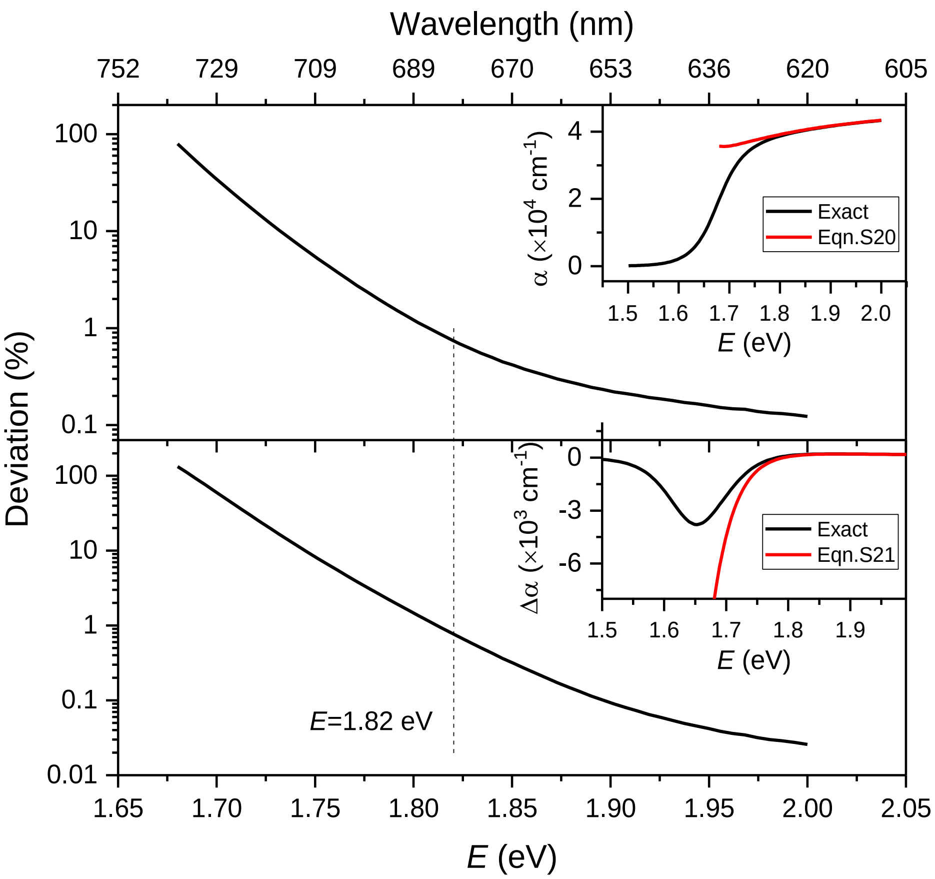

Supplementary Figure 6: Top panel: The high-energy part of the steady-state absorption spectrum can be approximated by neglecting the broadening function. Bottom panel: The high-energy part of the TA spectrum can be approximated by neglecting the broadening function. Parameters are: E_g=1.6769 eV, R_{b0}=11.3 meV, ΔR_b=1 meV, ΔE_{bgr}=-5 meV, T_e=350 K, E_F=1.62 eV. Insets show comparisons between the 'exact' spectrum and the spectrum calculated using our simplified equations.

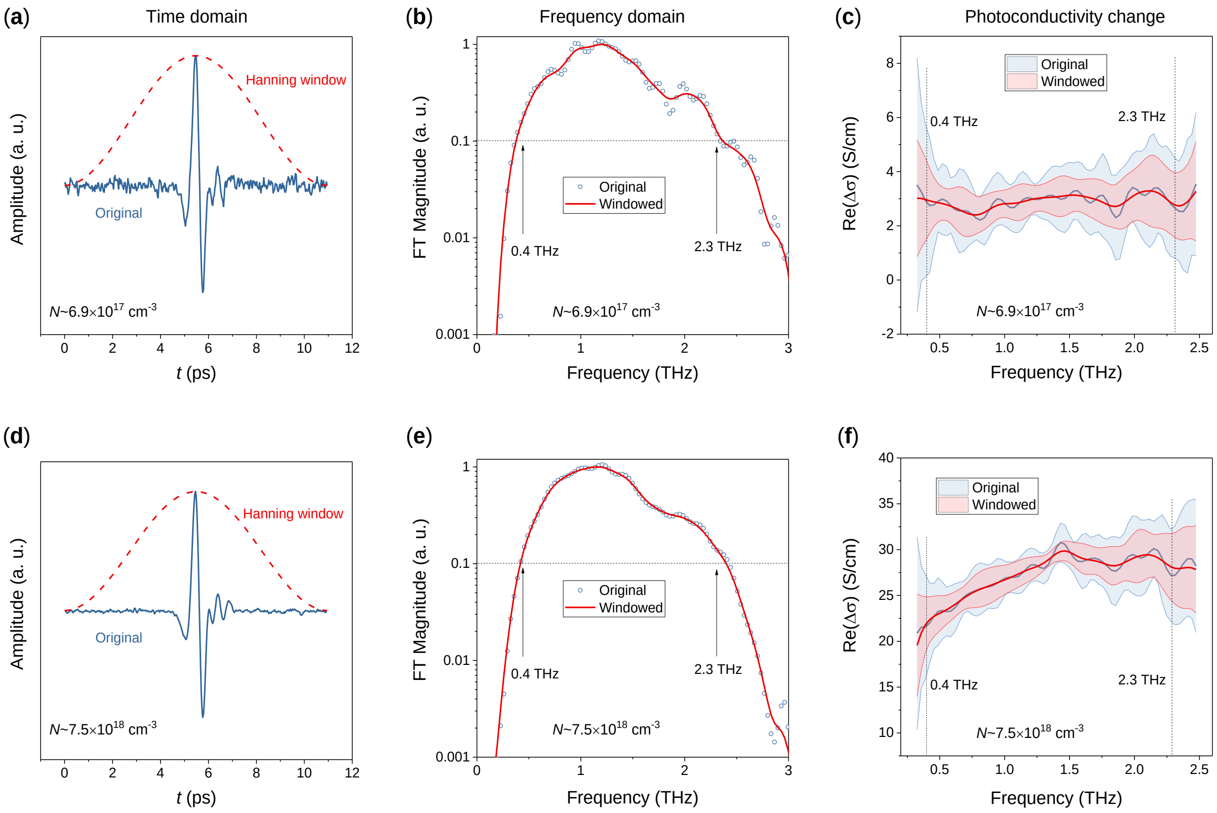

Supplementary Figure 7: (**a**) Photo-induced time-domain THz signal of CsFAMA thin film for a carrier density of ~6.9×10^{17} cm^{-3}. The time interval is 30 fs. The red dashed line indicates the FT window (Hanning). (**b**): FT magnitude of the original signal (blue open circles) and of the windowed signal (red solid line), respectively. The normalized FT magnitude in the frequency range from 0.4~2.3 THz is larger than 0.1. (**c**) Photo-induced change of photoconductivity (solid lines) and uncertainty (standard error, filled areas) determined from multiple measurements. The spectra between 0.4~2.3 THz exhibit less error, so this frequency range was selected for all THz spectra in this work. (**d-f**) The same spectra as (**a-c**) for a carrier density of ~7.5×10^{18} cm^{-3}.

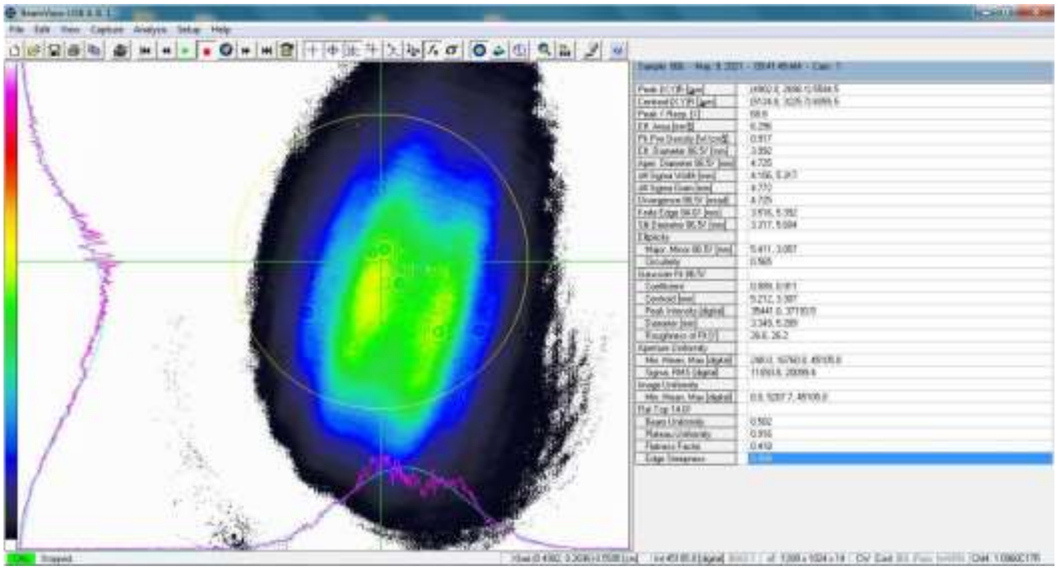

Supplementary Figure 8: The diameter of our THz experiment pump beam measured by a beam profiler.

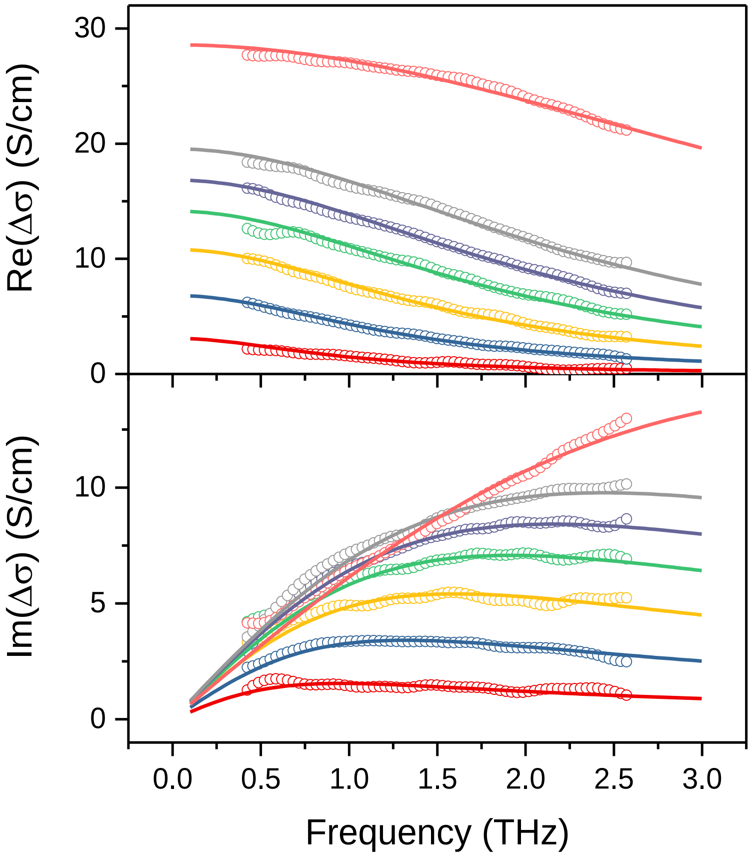

N, cm^-3	Wp, rad/ps	Wp err	tao, fs	tao err
2.74066E16	14.4946	0.29	166.5815	9.6389
7.42834E16	25.2605	0.219	120.6915	2.7553
1.44488E17	35.0986	0.1915	99.2201	1.3572
2.18383E17	43.8315	0.1865	83.1777	0.8525
2.73994E17	50.8032	0.1862	73.7078	0.6346
3.48181E17	58.1263	0.1901	65.343	0.4906
7.4471E17	94.826	0.2894	35.9019	0.2318

Supplementary Figure 9: Δσ of a n-doped silicon wafer (resistivity ~1-3 Ω, thickness ~283 μm) at different pump fluence. Solid lines show Drude fits to the data, indicating that our THz experiment is free of pump size effects. The fit results are shown in the table.

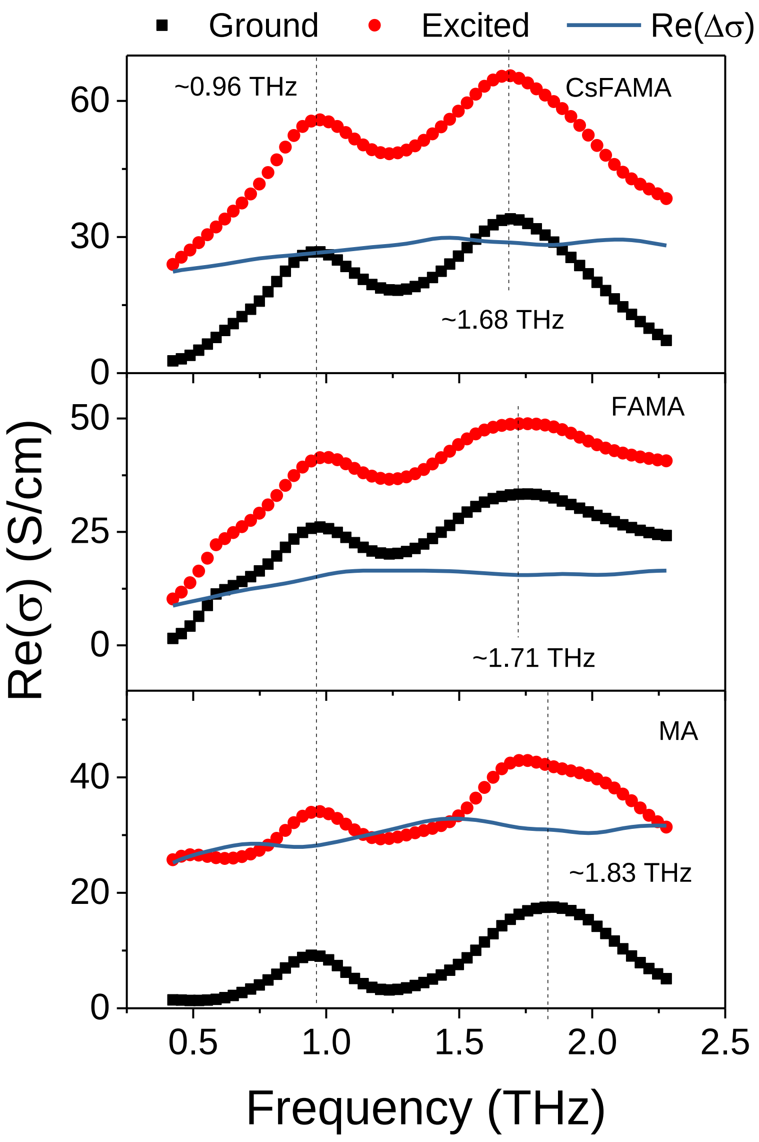

Supplementary Figure 10: The real part of the THz photoconductivity in the ground state and excited state for CsFAMA, FAMA and MA thin films, respectively. Blue lines indicate the difference spectra ($\Delta\sigma$). No obvious peak shift and shape change of TO-phonon resonances are observed in our samples, except a small weakening of the resonance amplitude.

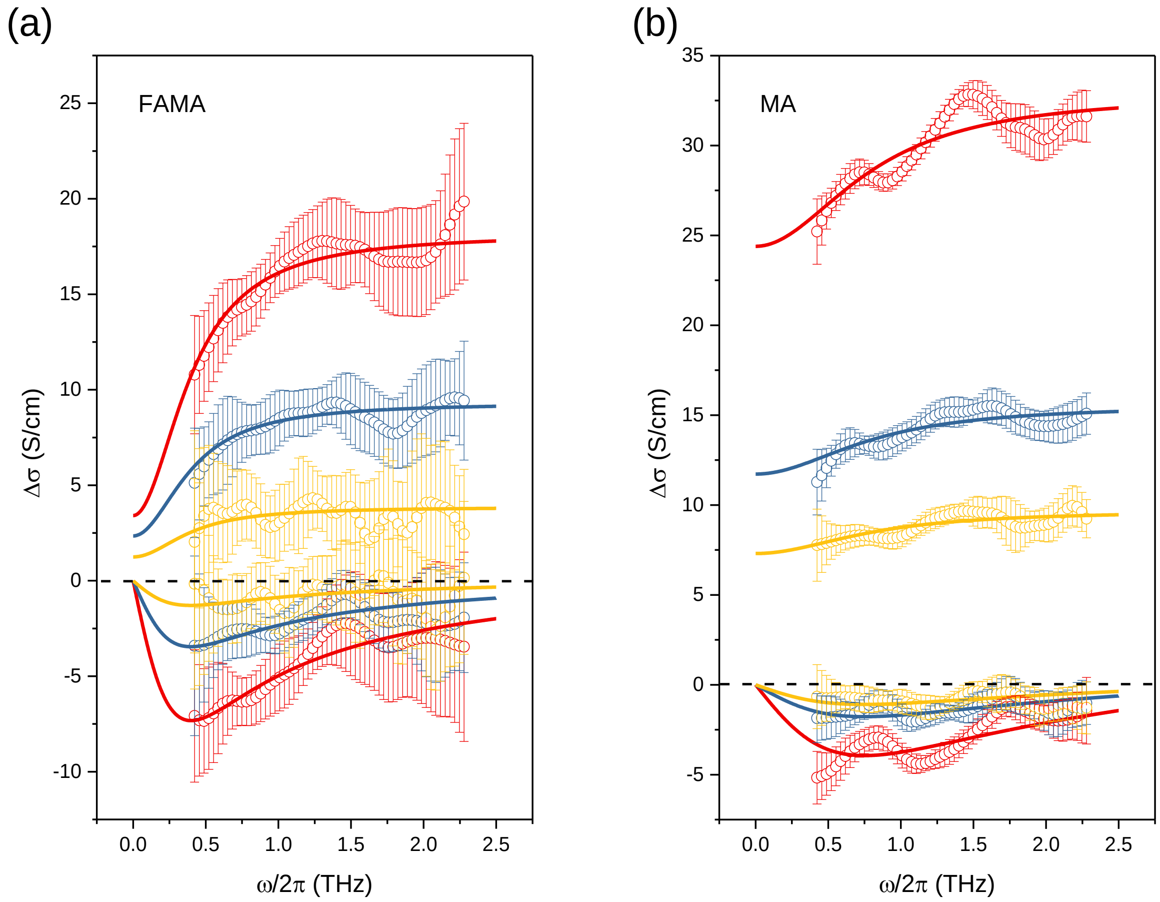

Supplementary Figure 11: The Drude-Anderson fits (solid lines) to the photo-induced THz photoconductivity spectra (Δσ) of FAMA (**a**) and MA (**b**), respectively. The negative curves correspond to Im(Δσ). The fit results are provided in Tab. S2 and S3.

Supplementary Table 1: Fit parameters of the THz photoconductivity change of CsFAMA thin film sample.

	Method	Drude-Debye	Drude-Smith	Drude-Anderson	Drude-Lorentz
	Explanation	Phonon resonance	Localization	Quantum localization	Energy barrier resonance
CsFAMA<br>~300 nm<br><br>Cs:0.05<br>FA:0.8<br>MA:0.15<br>I:0.85<br>Br:0.15<br><br>N (cm^{-3}):<br>7.49×10^{18}<br>3.43×10^{18}<br>2.437×10^{18}<br>1.787×10^{18}<br>1.02×10^{18}<br>6.885×10^{17}	R-square	0.9978	0.9946	0.9978	0.9979
	τ_s (fs)<br>Polaron scattering time<br>Or $\phi_b \tau_s$<br>Diffuse time	1.0419±0.0183<br>1.0758±0.021<br>1.2125±0.0193<br>1.2694±0.0193<br>1.321±0.025<br>1.4654±0.0339	1.5906±0.0077<br>1.5888±0.0117<br>1.7035±0.0103<br>1.6545±0.0128<br>1.759±0.0154<br>1.7635±0.0256	1.7592±0.0067<br>1.7361±0.0107<br>1.8816±0.0093<br>1.7894±0.0112<br>1.9484+0.0136<br>1.9002±0.0216	1.3076±0.0106<br>1.3348±0.0143<br>1.5128±0.0112<br>1.5025±0.0125<br>1.6179±0.0159<br>1.6782±0.0225
	ϕ<br>Fraction of localized carriers<br>$\phi\phi_b$ *or* c_j<br>Localization level	/	-0.1836±0.004<br>-0.1795±0.0074<br>-0.1712±0.0058<br>-0.1405±0.0078<br>-0.149±0.01<br>-0.1107±0.0151	0.4054±0.0084<br>0.377±0.0108<br>0.3541±0.0088<br>0.288±0.0101<br>0.321±0.0126<br>0.228±0.0186	0.2233±0.0076<br>0.1962±0.0085<br>0.1676±0.0056<br>0.1313±0.0058<br>0.1436±0.0071<br>0.0962±0.0089
	j<br>Backscattering at j-th scattering	/	121.4663±2.2569	/	/
	σ_0 (S/cm)<br>Photo-induced increase of dielectric response	12.289±0.257<br>5.17±0.157<br>3.718±0.097<br>2.124±0.079<br>1.462±0.061<br>0.684±0.059	/	/	/
	τ_r (fs)<br>Debye relaxation time	261.045±9.119	/	/	/
	$\phi_b \tau_b$ (fs)<br>Backscattering time	/	/	245.694±7.942	/
	L (nm)<br>Localization length	/	~3.36-3.72	~3.98-4.14	/
	$\omega_0/2\pi$ (THz)<br>Center frequency of resonance	~ 3.9-9.5 THz<br>~16-39 meV<br>~130-390 cm^{-1}	/	/	~5.52±0.05 THz<br>~22.63±0.21 meV<br>~184±1.7 cm^{-1}
	τ_0 (fs)<br>Damping time of resonance	$\tau_s < \tau_0 < 6.37$	/	/	$\tau_s \exp(E_h/k_B T)$<br>~3.2-4.1

Localization length: $L \cong \sqrt{\tau_s \tau_b}\sqrt{2k_B T/m^*}$

Errors correspond to the 95% confidence interval.

Supplementary Table 2: Fit parameters of the THz photoconductivity changes of FAMA thin film sample.

	Method	Drude-Debye	Drude-Smith	Drude-Anderson	Drude-Lorentz
	Explanation	Phonon resonance	Localization	Quantum localization	Energy barrier resonance
FAMA<br>~300 nm<br><br>FA:0.83<br>MA:0.17<br>I:0.83<br>Br:0.17<br><br>N (cm^{-3}):<br>6.8×10^{18}<br>3.2×10^{18}<br>1.32×10^{18}	R-square	0.9937	0.9732	0.9937	0.9934
	τ_s (fs)<br>Polaron scattering time	0.2156±0.0438<br>0.3218±0.0480<br>0.4083±0.0837	0.9969±0.0133<br>1.0481±0.0179<br>1.0688±0.0473	1.1396±0.0087<br>1.2414±0.0107<br>1.2462±0.0314	0.5431±0.0348<br>0.7848±0.0252<br>0.8496±0.0638
	ϕ<br>Fraction of localized carriers<br>$\phi\phi_b$ *or* c_j<br>Localization level	/	-0.2951±0.0111<br>-0.2742±0.0169<br>-0.2174±0.0560	0.8121±0.0358<br>0.7481±0.0367<br>0.6767±0.0657	0.5880±0.0646<br>0.335±0.0244<br>0.2744±0.050
	j<br>Backscattering at j-th scattering	/	225.3148±8.0791	/	/
	σ_0 (S/cm)<br>Photo-induced increase of dielectric response	14.633±0.602<br>6.855±0.033<br>2.578±0.263	/	/	/
	τ_r (fs)<br>Debye relaxation time	405.224±23.154	/	/	/
	$\phi_b\tau_b$ (fs)<br>Backscattering time	/	/	395.993±21.665	/
	L (nm)<br>Localization length	/	~2.86-3.07	~4.07-4.26	/
	$\omega_0/2\pi$ (THz)<br>Center frequency of resonance	~ 3.1-17 THz<br>~12.8-69.8 meV<br>~104-567 cm^{-1}	/	/	~6.39±0.04 THz<br>~26.2±0.16 meV<br>~213±1.3 cm^{-1}
	τ_0 (fs)<br>Damping time of resonance	$\tau_s < \tau_0 < 6.37$	/	/	$\tau_s\exp(E_h/k_BT)$<br>~0.696-1.089

Localization length: $L \cong \sqrt{\tau_s\tau_b}\sqrt{2k_BT/m^*}$

Errors indicate the 95% confidence interval.

Supplementary Table 3: Fit parameters of the THz photoconductivity changes of MA thin film sample.

	Method	Drude-Debye	Drude-Smith	Drude-Anderson	Drude-Lorentz
	Explanation	Phonon resonance	Localization	Quantum localization	Energy barrier resonance
MA<br>~300nm<br><br>MA: 1<br>I: 1<br><br>N (cm^{-3}):<br>6.8×10^{18}<br>3.18×10^{18}<br>1.9×10^{18}<br>1.2×10^{18}	R-square	0.9971	0.9966	0.9972	0.9973
	τ_s (fs)<br>Polaron scattering time	1.5365±0.02<br>1.5781±0.024<br>1.6456±0.032<br>1.6599±0.06	1.8715±0.0068<br>1.8821±0.0111<br>1.9556±0.0152<br>1.9346±0.0307	2.0661±0.0103<br>2.091±0.015<br>2.177±0.0214<br>2.1262+0.041	1.7398±0.0114<br>1.7816±0.0155<br>1.8697±0.0209<br>1.8536±0.0407
	ϕ<br>Fraction of localized carriers<br>$\phi\phi_b$ *or* c_j<br>Localization level	/	-0.1356±0.0029<br>-0.1252±0.0056<br>-0.1262±0.0092<br>-0.1129±0.0199	0.2594±0.0067<br>0.248±0.0099<br>0.2468±0.015<br>0.2218±0.0315	0.1225±0.005<br>0.1144±0.0062<br>0.1114±0.0084<br>0.099±0.0165
	j<br>Backscattering at j-th scattering	/	114.8249±1.9221	/	/
	σ_0 (S/cm)<br>Photo-induced increase of dielectric response	8.137±0.218<br>3.701±0.161<br>2.285±0.153<br>1.27±0.199	/	/	/
	τ_r (fs)<br>Debye relaxation time	205.885±11.895	/	/	/
	$\phi_b\tau_b$ (fs)<br>Backscattering time	/	/	196.741±10.196	/
	L (nm)<br>Localization length	/	~3.84-3.96	~3.86-3.91	/
	$\omega_0/2\pi$ (THz)<br>Center frequency of resonance	~ 4.4-8.9 THz<br>~18-36.6 meV<br>~146-298 cm^{-1}	/	/	~5.47±0.08 THz<br>~22.4±0.33 meV<br>~182±2.6 cm^{-1}
	τ_0 (fs)<br>Damping time of resonance	$\tau_s<\tau_0<6.37$	/	/	$\tau_s\exp(E_h/k_BT)$<br>~4.26-4.48

Localization length: $L \cong \sqrt{\tau_s\tau_b}\sqrt{2k_BT/m^*}$

Errors indicate the 95% confidence interval.

Supplementary Section 1: Ground-state absorption coefficient

Ground-state absorption measurements were performed using a PerkinElmer Lambda 950 UV/Vis/NIR spectrophotometer. To minimize the impact of reflection of light on the absorption spectra, the absorption coefficient is usually calculated by:

$$\alpha(E) = -\frac{1}{d}\ln\left(\frac{T}{1-R}\right) \qquad (S1)$$

where *d* is the sample thickness, *R* is the reflectance, and *T* is the transmittance. However, this equation is not sufficiently accurate for thin films, since it neglects internal reflections. Hence, we used a modified equation to extract the absorption spectra:

$$\alpha(E) = -\frac{1}{d}\ln\left(\sqrt{a^2 + \frac{1}{1-b-bc}} - a\right), \qquad a = \frac{b(1-R+cT)}{2(1-b-bc)T} \qquad (S2a)$$

$$\alpha\left(E \geq E_{opt}\right) = -\frac{1}{d}\ln\frac{T}{b(1-R+cT)} \qquad (S2b)$$

where n_2 is the averaged refractive index of perovskite films in the measured wavelength window. $b = \frac{16n_2^2 n_s}{(n_2+n_s)^2(n_s+1)^2}$ and $c = \frac{(n_s-1)^2}{4n_s}$ are constants that account for the internal reflections and coherent light propagation. b~0.9 and c~0.042 are obtained when using n_2=2.5 and n_s=1.5.

R and *T* used in the evaluation of Eqn. S2 were calculated from the reported complex refractive index of MAPbI [Ref. S1]. *R* and *T* in a three-media system (3M) involved with coherent propagation are given by:

$$R_{3M} = |r^2| = \left|r_{12} + \frac{t_{12}r_{23}t_{21}e^{-(\alpha+2i\beta)d}}{1 - r_{21}r_{23}e^{-(\alpha+2i\beta)d}}\right|^2 = \left|\frac{r_{12} + r_{23}e^{-(\alpha+2i\beta)d}}{1 + r_{12}r_{23}e^{-(\alpha+2i\beta)d}}\right|^2 \qquad (S3a)$$

$$T_{3M} = \frac{n_3}{n_1}|t^2| = \frac{n_3}{n_1}\left|\frac{t_{12}t_{23}e^{-\left(\frac{\alpha}{2}+i\beta\right)d}}{1 - r_{21}r_{23}e^{-(\alpha+2i\beta)d}}\right|^2 = T_{12}T_{23}e^{-\alpha d}\left|1 + r_{12}r_{23}e^{-(\alpha+2i\beta)d}\right|^{-2} \qquad (S3b)$$

where 1, 2, and 3, respectively, refer to the air, the perovskite, and the substrate. *r* and *t* are the reflectance and transmittance, respectively. Specifically, $r_{12} = \frac{\tilde{n}_1-\tilde{n}_2}{\tilde{n}_1+\tilde{n}_2}, t_{12} = \frac{2\tilde{n}_1}{\tilde{n}_1+\tilde{n}_2}$, where $\tilde{n} = n + ik$ is the complex refractive index. $\alpha=4\pi k_2 d/\lambda$ and $\beta=2\pi n_2 d/\lambda$ are, respectively, the absorption coefficient and the phase constant that account for coherence. For a quartz substrate of finite thickness (1 mm in our case) we find that the media can be considered as optically thick so that the propagation becomes incoherent. Thus, we can derive the entire expression for *R* and *T*:

$$R = R_{3M} + \frac{T_{3M}R_{31}T'_{3M}}{1 - R'_{3M}R_{31}} \quad (S4a)$$

$$T = \frac{T_{3M}T_{31}}{1 - R'_{3M}R_{31}} \quad (S4b)$$

where R'_{3M} and T'_{3M}, respectively, represent the reflectance and transmittance of inverse propagation in the 3M system. Their value can be calculated by Eqn. S3 when swapping subscript 1 and 3. $R_{31} = \left(\frac{n_3 - n_1}{n_3 + n_1}\right)^2$ and $T_{31} = 1 - R_{31}$, respectively, represent the reflectance and transmittance at the interface of substrate and air.

The calculated R and T values when using Eqn. S4 are plotted in **Supplementary Figure 1a**. The exact α and simulated α determined from Eqn. S1 and S2 are plotted in **Supplementary Figure 1b**. The difference spectra (**Supplementary Figure 1c**) indicate that the error from Eqn. S2 is smaller than 2% above the optical bandgap (E_{opt}~1.6 eV). The validation of Eqn. S2 comes from the fact that $n >> k$ and the dispersion of n is small in metal-halide perovskites. T_{3M} can be simplified from Eqn. S3b when using $n_2 >> k_2$ and a constant n_2:

$$R_{3M} = 1 + \frac{T_{12}R_{23}e^{-2\alpha d} - T_{12}}{1 + r_{12}r_{23}e^{-\alpha d}\cos 2\beta d + R_{12}R_{23}e^{-2\alpha d}} \quad (S5a)$$

$$T_{3M} = \frac{T_{12}T_{23}e^{-\alpha d}}{1 + r_{12}r_{23}e^{-\alpha d}\cos 2\beta d + R_{12}R_{23}e^{-2\alpha d}} \quad (S5b)$$

Applying the double-reflection model to the substrate:

$$R \cong R_{3M} + T_{3M}R_{31} = 1 + \frac{T_{12}R_{23}e^{-2\alpha d} - T_{12}}{1 + r_{12}r_{23}e^{-\alpha d}\cos 2\beta d + R_{12}R_{23}e^{-2\alpha d}} + \frac{R_{31}}{T_{31}}T \quad (S6a)$$

$$T \cong T_{3M}T_{31} = \frac{T_{12}T_{23}T_{31}e^{-\alpha d}}{1 + r_{12}r_{23}e^{-\alpha d}\cos 2\beta d + R_{12}R_{23}e^{-2\alpha d}} \quad (S6b)$$

Next, the coherent terms cancel:

$$R \cong 1 + \frac{R_{31}}{T_{31}}T + \frac{R_{23}e^{-2\alpha d} - 1}{T_{23}T_{31}e^{-\alpha d}}T \quad (S7a)$$

$$e^{-\alpha d} \cong -\frac{T_{23}T_{31}\left(1 - R + \frac{R_{31}}{T_{31}}T\right)}{2R_{23}T} + \sqrt{\frac{T_{23}^2T_{31}^2\left(1 - R + \frac{R_{31}}{T_{31}}T\right)^2}{4R_{23}^2T^2} - \frac{1}{R_{23}}} \quad (S7b)$$

Eqn. S2a can be derived when setting $b = T_{23}T_{31}$ and $c = R_{31}/T_{31}$. A simpler expression (Eqn. S2b) can be obtained by neglecting the higher-order absorption term in Eqn. S7a ($e^{-2\alpha d} \rightarrow 0$). This treatment increases the derivation below E_{opt} due to weak absorption (**Supplementary Figure 1c**).

Supplementary Section 2: Transient absorption (TA) spectroscopy

Our TA setup uses a commercial Ti:sapphire amplifier operating at 800 nm with a repetition rate of 3 kHz as laser source. Its pulse width (FWHM) is compressed to ~125 fs. Two optical parametric amplifiers (OPA) are used to tune the laser wavelength. The white-light probe is generated by 1300 nm laser (from TOPAS1) with a CaF2 crystal that mounted on a continuously moving stage, which enables us to generate a super-continuum pulses with a spectral range from 350 to 1100 nm. The pump laser (from TOPAS2) is chopped to 1.5 KHz and delayed by an automated mechanical delay stage (Newport linear stage IMS600CCHA) from -400 ps to 8 ns. Pump and probe beams were overlapped on the front surface of the sample, and their spot sizes (D86~3 mm) were measured by a beam viewer (Coherent, LaserCam-HR II) to make sure the pump beam was about three times larger than the probe beam (D86~1 mm). The perovskite samples are stored in a nitrogen-filled chamber to protect from degradation, and photo-excited by 475 nm, 550 nm, and 675 nm in this work. The probe beam was guided to a custom-made prism spectrograph (Entwicklungsbüro Stresing) where it was dispersed by a prism onto a 512 pixel complementary metal-oxide semiconductor (CMOS) linear image sensor (Hamamatsu G11608-512DA). In order to account for the reflection, we first measured the transient reflection ($\Delta R/R$), then measured the transient absorption ($\Delta T/T$).

Since Eqn. S2b derived from Eqn. S7a by neglecting the higher-order term provides a decent approximation of α above the optical bandgap, we start from Eqn. S2b to derive the relation between $\Delta\alpha$ and the measured $\Delta R/R$, $\Delta T/T$:

$$f = \frac{T}{1 - R + cT} = b\, e^{-\alpha d} \qquad (S8)$$

Taking the total derivative and noticing that c is a constant and b is not sensitive to $\Delta\alpha$:

$$\frac{\Delta f}{\Delta\alpha} = \frac{\partial f}{\partial\alpha} + \frac{\partial f}{\partial n_2}\frac{\Delta n_2}{\Delta\alpha} \rightarrow\ \Delta f = \frac{\partial f}{\partial\alpha}\Delta\alpha + \frac{\partial f}{\partial n_2}\Delta n_2\ \rightarrow$$

$$\frac{\Delta T}{T} - \frac{\Delta R - c\Delta T}{1 - R + cT} = -\Delta\alpha d - \frac{(n_2 - n_3)\Delta n_2}{n_2(n_2 + n_3)} \qquad (S9)$$

The second term at right side is small:

$$\frac{(n_2 - n_3)\Delta n_2}{n_2(n_2 + n_3)} \xrightarrow{\text{KK relation}} \sim \frac{(n_2 - n_3)\Delta k_2}{n_2(n_2 + n_3)} \cong \frac{3\lambda}{80\pi d}\Delta\alpha d \ll \Delta\alpha d \qquad (S10)$$

Because c is small (<0.05), the photo-induced change of the absorption coefficient can be expressed as:

$$\Delta\alpha(E) \cong -\frac{1}{d}\left(\frac{\Delta T}{T} - \frac{\Delta R - c\Delta T}{1 - R + cT}\right) \cong -\frac{1}{d}\left(\frac{\Delta T}{T} - \frac{\Delta R/R}{1/R_0 - 1}\right) \qquad (S11)$$

where R_0 is the ground-state reflectivity spectrum. For large $\Delta\alpha$, Eqn. S9 is no longer valid, hence $\Delta\alpha$ can be calculated by:

$$\Delta\alpha(E) = -\frac{1}{d}\ln\frac{T + \Delta T}{b[1 - (R + \Delta R) + c(T + \Delta T)]} - \left[-\frac{1}{d}\ln\frac{T}{b(1 - R + cT)}\right]$$

$$= -\frac{1}{d}\ln(1 + \Delta T/T) - \frac{1}{d}\ln\frac{1 - (R + \Delta R) + c(T + \Delta T)}{1 - R + cT}$$

$$\cong -\frac{1}{d}\ln\frac{1 + \Delta T/T}{1 - \frac{\Delta R/R}{1/R_0 - 1}} \qquad (S12)$$

The same expression of Eqn. S12 can be derived from Eqn. S1, indicating that internal reflections hardly affect the photo-induced change of α in perovskite thin films deposited on quartz substrates. Since Eqn. S12 is equivalent to S11 for small $\Delta\alpha$, all the $\Delta\alpha$ in this work were calculated by Eqn. S12. **Supplementary Figure 4c** is an example of the data processing.

Supplementary Section 3: Determination of the carrier density and thermal effect

The carrier densities generated by photoexcitation were calculated by:

$$N = F_{ph} \cdot \alpha_{pump} \qquad (S13)$$

where α_{pump} is the absorption coefficient at pump photon energy (E_{ph}). F_{ph} is the photon flux calculated by deducting the fraction of surface-reflected photons from the total number of incident photons:

$$F_{ph} = \frac{P(1 - R_{pump})}{1500\ Hz \cdot \pi \cdot (D/2)^2 \cdot E_{ph}} \qquad (S14)$$

where P is the pump power and R_{pump} is the reflectivity at E_{ph},. The factor 1500 denotes the pump laser pulse repetition rate in Hz. D represents the beam diameter of the pump beam measured by the beam profiler for the TA experiments (~3 mm, D86) and for the THz experiments (~4 mm, D86).

The heat accumulation effect can be ruled out in our experiments, as mentioned in the main text. The temperature rise caused by a single excitation pulse is smaller than 0.32 K for a carrier density of 3.5×10^{18} cm^{-3} (i.e., the highest carrier density used in our TA experiments) when using 550 nm photons (~2.25 eV), calculated using the heat capacity of the MHP lattice (~170-190 J/K/mol at room temperature [Ref. S2]):

$$\Delta T_l = \frac{\Delta E_{ex} NV}{C_{MHP} * Mol} = \frac{(2.25\, eV - 1.6\, eV) * 1.6 * 10^{-19}\, J/eV * 3.5e18\, cm^{-3} * 1\, cm^3}{170 \frac{J}{K \cdot mol} \cdot \frac{4e21}{N_A} mol} \cong 0.32\, K \quad (S15)$$

where ΔE_{ex} is the excess energy of the pump photons. N is carrier density. V is the volume in cm^3. C_{MHP} is the molar heat capacity of the MHP lattice, where we used the lower-bound value of 170 J/K/mol. Here, 4×10^{21} is the number of unit cells in V=1 cm^3. N_A is the Avogadro constant.

Supplementary Section 4: Elliott fit to the absorption spectra

The ground-state absorption coefficient spectra were fitted by a convolution of broadening functions (Eqn. S18) and the following Elliott formula:

$$\alpha(E) = A \frac{4\pi R_{b0}^{3/2}}{E} \delta(x + R_{b0}) + A \frac{\xi(R_{b0}, x)}{E} \sqrt{x} \quad (S16)$$

where

$$\xi(R_{b0}, x) = \frac{2\pi\sqrt{R_{b0}/x}}{1 - \exp\left(-2\pi\sqrt{R_{b0}/x}\right)}, \qquad x = E - E_g \quad (S17)$$

Here, we evaluated three different broadening functions in the fit: Gaussian, hyperbolic-secant, and Voight (Gaussian convoluted with Lorentzian). Both the hyperbolic-secant distribution and Voight distribution result in excellent fits (**Supplementary Figure 2**). Since the hyperbolic-secant has only one parameter, we finally choose the hyperbolic-secant distribution as broadening function due to its simplicity.

$$Gaussian:\ G(E, \sigma) = \frac{1}{\sqrt{2\pi}\sigma} \exp\left(-\frac{E^2}{2\sigma^2}\right) \quad (S18a)$$

$$hyperbolicsecant:\ S(E, \sigma) = \frac{1}{2\sigma} \operatorname{sech}\left(\frac{\pi E}{2\sigma}\right) \quad (S18b)$$

$$Voight:\ V(E, \sigma, \sigma_{lo}) = G(E, \sigma) \otimes \frac{\sigma_{lo}}{\pi} \frac{1}{E^2 + \sigma_{lo}^2} \quad (S18c)$$

The convolution of the continuum absorption and an arbitrary broadening function is:

$$\frac{A}{E}\xi(R_{b0},x)\sqrt{x}\otimes G(E,\sigma)=\frac{A}{E}\int_{-\infty}^{+\infty}\xi(R_{b0},t)\sqrt{t}S(x-t,\sigma)dt \qquad (S19)$$

If $x>>\sigma$, then the broadening function is relatively narrow and we can consider $\xi(R_{b0}, t)t^{1/2}$ as varying weakly. Thus, the integral can be avoided and we obtain:

$$\frac{A}{E}\xi(R_{b0},x)\sqrt{x}\otimes S(E,\sigma)\approx\frac{A}{E}\xi(R_{b0},x)\sqrt{x} \qquad (S20)$$

The deviation of this equation is smaller than 1% at E~1.8 eV for our FAMACs sample (top panel of **Supplementary Figure 6**). Therefore, the photo-induced change of the absorption coefficient can be calculated by:

$$\Delta\alpha(E)\cong\frac{A}{E}\xi\left(R_{b0}-\Delta R_b,x-\Delta E_{bgr}\right)\sqrt{x-\Delta E_{bgr}}(1-f_e)^2-\frac{A}{E}\xi(R_{b0},x)\sqrt{x} \qquad (S21)$$

where ΔR_b and ΔE_{bgr} are the screened exciton binding energy and the photo-induced BGR due to many-body effects, respectively. Here a negative ΔE_{bgr} represents bandgap narrowing. $f_e=1/[1+e^{(E-E_F)/k_BT_e}]$ is the Fermi-Dirac distribution function accounting for the occupation probability of electrons in the conduction band, where E_F is the quasi-Fermi level, k_B is the Boltzmann constant, and T_e is the absolute carrier temperature. Since the effective mass of holes is similar to that of electrons, we can assume that the occupation probability of holes in the valence band is symmetric to that of electrons in the conduction band.

When E-E_F is much larger than k_BT_e, then it satisfies $f_e<<1$, and Eqn. S21 can be further simplified. Applying a Taylor expansion and neglecting higher order terms we obtain:

$$\sqrt{x-\Delta E_{bgr}}(1-f_e)^2\cong\sqrt{x}\left(1-\frac{\Delta E_{bgr}}{2x}\right) \qquad (S22a)$$

$$\xi\left(R_b-\Delta R_b,x-\Delta E_{bgr}\right)\cong\xi_0\left[1-\left(1-\xi_0e^{-2\pi\sqrt{\frac{R_{b0}}{x}}}\right)\left(\frac{\Delta R_b}{2R_{b0}}-\frac{\Delta E_{bgr}}{2x}\right)\right] \qquad (S22b)$$

where ξ_0 represents $\xi(R_{b0}, x)$. Consequently, the relation between $\Delta\alpha$ and ΔE_{bgr} can be approximated as:

$$\Delta\alpha(E)\cong\frac{A}{E}\xi_0\sqrt{x}\left[\left(\xi_0e^{-2\pi\sqrt{\frac{R_{b0}}{x}}}-1\right)\frac{\Delta R_b}{2R_{b0}}-\xi_0e^{-2\pi\sqrt{\frac{R_{b0}}{x}}}\frac{\Delta E_{bgr}}{2x}\right] \qquad (S23)$$

Supplementary Section 5: Time-domain terahertz spectroscopy setup (td-THz)

Our td-THz setup uses the same Ti:sapphire amplifier as the TA setup. The THz emitter and detector are two 1 mm thick <110> oriented zinc telluride (ZnTe) crystals. All the THz related

optics were placed in a closed chamber, which was continuously purged with pure nitrogen gas. Perovskite samples were excited by 550 nm laser pulses obtained from the same TOPAS2 as used in the TA experiment. A motor equipped with a circular ND filter was used to change the pump fluence in the fluence dependent experiments.

The Hanning window was applied to the time-domain THz spectra to minimize the effect of frequency leakage caused by the discontinuity at boundaries. For terahertz waveforms with pulse-like signals, the Hanning window is a good choice to resolve the frequency of interest by (1) removing boundary discontinuity in the time domain and (2) smoothing the broadband random noise in the frequency domain (**Supplementary Figure 7**).

Supplementary Section 6: Examination of the size effect in the THz spectra

Assuming that the terahertz electric field is Gaussian, the spot size of the terahertz probe can be calculated from the ratio of the maximum terahertz field transmitted through a 1-mm pinhole to its original terahertz field:

$$T_{max} = \frac{E_{1\text{mm}}}{E_0} = \frac{\iint_{\theta=0,\ r=0}^{\theta=2\pi,\ r=1\text{ mm}} e^{-\frac{r^2}{w^2}} r d\theta dr}{\iint_{\theta=0,\ r=0}^{\theta=2\pi,\ r=\infty} e^{-\frac{r^2}{w^2}} r d\theta dr} = 1 - e^{-\frac{1\text{ mm}^2}{w^2}} \qquad (S24a)$$

$$D_{THz} = 2w = \sqrt{-\frac{4}{\ln(1-T_{max})}} \quad \text{mm} \qquad (S24b)$$

The diameter (D86) of the THz probe is ~2.4 mm as calculated from the measured T_{max} ~50%. We set the pump size to ~4 mm (D86, **Supplementary Figure 8**) to avoid any spot-size effects [Ref. S3] and to maximize the photon fluence. **Supplementary Figure 9** shows that our THz experiment is free of spot size effects.

Supplementary Section 7: Optical phonon absorption in the THz spectra

The optical phonon effects on the photo-induced change of the photoconductivity spectra ($\Delta\sigma$) are mainly from the infrared-active TO-phonons:

a. Artifact due to data analysis:

$$\frac{\Delta E_{THz}}{E_{THz}} = -\frac{1}{\varepsilon_0 c(1+n_{sub})} \int_0^d \Delta\sigma(\omega, x) dx \qquad (S25)$$

The equation above is derived based on the zeroth-order approximation [Ref. S4] that exhibits errors at frequencies with high dielectric background, yielding an artifact in the calculated $\Delta\sigma$, especially at the peak of the TO-phonon resonance. Nevertheless, such artifact has a small

influence on the Δσ's amplitude, and thus the frequency dependence in metal-halide perovskites [Ref. S4].

b. Photo-induced change of TO-phonon resonances:
   Photo-induced peak-shifts of the TO-phonon resonances were reported in CsPbBr nanocrystal thin film [Ref. S5] and ascribed to polaronic effects. However, no obvious photo-induced peak shift and shape change of the TO-phonon resonances was observed in our samples (**Supplementary Figure 10**), except a small weakening of the resonance amplitude, which is typically caused by thermal expansion of the TO-phonons. This observation evidences that the contribution of TO-phonons to Δσ is minor.

Supplementary Section 8: Models for a broadband Lorentz-like resonance

When a harmonic dipole oscillator is driven by the external field $E(t)$=E_0exp($-i\omega t$), the motion equation is:

$$\frac{d^2x}{dt^2}+\frac{1}{\tau_0}\frac{dx}{dt}+\omega_0^2x=\frac{eE(t)}{m^*} \qquad (S26)$$

where τ_0 and ω_0 are the damping time and center frequency of the oscillator, respectively. The analytical solution is Lorentzian:

$$x(t)=\frac{qE(t)}{m^*}\frac{i\tau_0}{\omega-i\tau_0(\omega^2-\omega_0^2)} \qquad (S27)$$

This motion adds a susceptibility:

$$\chi(\omega)=\frac{Nq^2}{\varepsilon_0 m^*}\frac{i\tau_0}{\omega-i\tau_0(\omega^2-\omega_0^2)} \qquad (S28)$$

where N is the dipole per volume. Then we have a Lorentzian resonance in the photoconductivity spectrum:

$$\sigma(\omega)=-i\varepsilon_0\omega\chi=\frac{N_V q^2}{\varepsilon_0 m^*}\frac{\omega\tau_0}{\omega-i\tau_0(\omega^2-\omega_0^2)} \qquad (S29)$$

***Drude response*:**

When $\omega_0 \rightarrow 0$, the Lorentzian resonance reduces to a Drude response, which describes the photoconductivity caused by free carrier scattering:

$$\sigma(\omega) = \frac{Nq^2}{\varepsilon_0 m^*}\frac{\tau_s}{1 - i\omega\tau_s} \tag{S30}$$

Drude-Lorentz response and Drude-Plasmon response

If the photoconductivity is caused by free carriers and the TO-phonon response, then the Drude-Lorentz model (DL model) can be applied:

$$\sigma(\omega) = \frac{Nq^2}{m^*}\left[\frac{\tau_s}{1 - i\omega\tau_s} - \frac{\omega\tau_0}{\omega - i\tau_0(\omega^2 - \omega_0^2)}\right] \tag{S31}$$

If carriers are partially localized with ϕ being the fraction of localized carriers, the DL model is:

$$\sigma(\omega) = \frac{Nq^2}{m^*}\left[\frac{(1-\phi)\tau_s}{1 - i\omega\tau_s} - \frac{\phi\omega\tau_0}{\omega - i\tau_0(\omega^2 - \omega_0^2)}\right], \tau_0 = \tau_s \exp\left(\frac{\hbar\omega_0}{k_B T}\right) \tag{S32}$$

If the localized carriers are surface plasmons, ω_0 can be replaced with the plasmon frequency:

$$\sigma(\omega) = \frac{Nq^2}{m^*}\left[\frac{(1-\phi)\tau_s}{1 - i\omega\tau_s} - \frac{\phi\omega\tau_0}{\omega - i\tau_0(\omega^2 - s\omega_p^2)}\right], \omega_p^2 = \frac{\phi Nq^2}{\varepsilon_0 m^*} \tag{S33}$$

where s=1/3 for three-dimensional localization [Ref. S6].

Debye relaxation

When the Lorentzian resonance is broad so that $\omega\tau_0 \ll 1$ is satisfied in the probed THz window, then the resonance can be approximated by Debye relaxation:

$$\sigma(\omega) \cong \frac{Nq^2}{\varepsilon_0 m^*}\frac{\omega\tau_0}{\omega + i\tau_0\omega_0^2} = -\frac{Nq^2}{\varepsilon_0 m^*}\frac{1}{\omega_0^2}\frac{i\omega}{1 - i\omega\tau_r} \tag{S34}$$

where $\tau_r = 1/(\omega_0^2\tau_0)$ is the relaxation time. If we assume that $\omega_{max}\tau_0 < 0.1$ and $\omega_{\mathrm{max}}/2\pi \sim 2.5$ THz for the THz observation window, then we obtain $\tau_0 < 6.37$ fs.

We obtain the Drude-Debye model (DD model) with ϕ being the fraction of localized carriers:

$$\Delta\sigma_{DD}(\omega) = \frac{Nq^2}{m^*}\left[\frac{(1-\phi)\tau_s}{1 - i\omega\tau_s} - \phi\tau_0\frac{i\omega\tau_r}{1 - i\omega\tau_r}\right] \tag{S35}$$

For the DD model without localized carriers we obtain:

$$\Delta\sigma_{DD}(\omega) = \frac{Nq^2}{m^*}\frac{\tau_s}{1-i\omega\tau_s} - \sigma_0\frac{i\omega\tau_r}{1-i\omega\tau_r} \tag{S36}$$

where σ_0 is the peak photoconductivity contributed by Debye relaxation. Since in this case the damping time τ_0 is unknown, the assumption of $\tau_s < \tau_0 < 6.37$ fs can be used to estimate the resonance peak by $\omega^2{}_0 = 1/(\tau_r\tau_0)$.

If $\omega\tau_s << 1$, then the two DD models are mathematically equivalent:

$$\frac{Nq^2}{m^*}\left[\frac{(1-\phi)\tau_s}{1-i\omega\tau_s} - \phi\tau_0\frac{i\omega\tau_r}{1-i\omega\tau_r}\right]$$

$$= \frac{Nq^2}{m^*}\frac{(1-\phi)\tau_s}{1-i\omega\tau_s} - \phi\tau_0\frac{Nq^2}{m^*}\frac{i\omega\tau_r}{1-i\omega\tau_r}$$

$$\cong \frac{Nq^2}{m^*}\frac{(1-\phi)\tau_s}{1-i\omega(1-\phi)\tau_s} - \phi\tau_0\frac{Nq^2}{m^*}\frac{i\omega\tau_r}{1-i\omega\tau_r}$$

$$= \frac{Nq^2}{m^*}\frac{\tau_s'}{1-i\omega\tau_s'} - \sigma_0\frac{i\omega\tau_r}{1-i\omega\tau_r}, \qquad \tau_s' = (1-\phi)\tau_s \tag{S37}$$

Drude-Anderson model mathematically equal to the Drude-Lorentz model if $\omega\tau_s$<<1

Considering the Drude-Anderson model (DA model) mentioned in the main article:

$$\Delta\sigma_{DA}(\omega) = \frac{Nq^2}{m^*}\left[\frac{\tau_s}{1-i\omega\tau_s} - \frac{\phi\phi_b\tau_s}{1-i\omega\phi_b\tau_b}\right]$$

$$= \frac{Nq^2}{m^*}\left[\frac{(1-\phi\phi_b)\tau_s}{1-i\omega\tau_s} + \frac{\phi\phi_b\tau_s}{1-i\omega\tau_s} - \frac{\phi\phi_b\tau_s}{1-i\omega\phi_b\tau_b}\right] \tag{S38}$$

If $\omega\tau_s << 1$, which is satisfied for MHPs in the THz region, then the DA model becomes mathematically equivalent to the DD model:

$$\Delta\sigma_{DA}(\omega) \cong \frac{Nq^2}{m^*}\left[\frac{(1-\phi\phi_b)\tau_s}{1-i\omega\tau_s} + \phi\phi_b\tau_s - \frac{\phi\phi_b\tau_s}{1-i\omega\tau_r}\right] \quad \tau_r = \phi_b\tau_b$$

$$= \frac{Nq^2}{m^*}\left[\frac{(1-\phi\phi_b)\tau_s}{1-i\omega\tau_s} - \phi\phi_b\tau_s\frac{i\omega\tau_r}{1-i\omega\tau_r}\right], \quad \tau_0 = \tau_s \tag{S39}$$

Since $\omega\tau_0$<<1 is satisfied by $\omega\tau_s$<<1 and $\tau_0=\phi_b\tau_s$, the DA model is mathematically equivalent to the DL model:

$$\Delta\sigma_{DA}(\omega) \cong \frac{Nq^2}{m^*}\left[\frac{(1-\phi\phi_b)\tau_s}{1-i\omega\tau_s} - \frac{\phi\phi_b\omega\tau_s}{\omega - i\tau_s(\omega^2-\omega_0^2)}\right], \omega_0^2 = \frac{1}{\phi_b\tau_s\tau_b} \qquad (S40)$$

Supplementary References

[S1] Phillips, L. J. *et al.* Dispersion relation data for methylammonium lead triiodide perovskite deposited on a (100) silicon wafer using a two-step vapour-phase reaction process. *Data in brief* **5,** 926–928; 10.1016/j.dib.2015.10.026 (2015).

[S2] Onoda-Yamamuro, N., Matsuo, T. & Suga, H. Calorimetric and IR spectroscopic studies of phase transitions in methylammonium trihalogenoplumbates (II)†. *Journal of Physics and Chemistry of Solids* **51,** 1383–1395; 10.1016/0022-3697(90)90021-7 (1990).

[S3] Beard, M. C., Turner, G. M. & Schmuttenmaer, C. A. Transient photoconductivity in GaAs as measured by time-resolved terahertz spectroscopy. *Phys. Rev. B* **62,** 15764–15777; 10.1103/PhysRevB.62.15764 (2000).

[S4] La-O-Vorakiat, C. *et al.* Phonon features in terahertz photoconductivity spectra due to data analysis artifact: A case study on organometallic halide perovskites. *Appl. Phys. Lett.* **110,** 123901; 10.1063/1.4978688 (2017).

[S5] Cinquanta, E. *et al.* Ultrafast THz Probe of Photoinduced Polarons in Lead-Halide Perovskites. *Phys. Rev. Lett.* **122,** 166601; 10.1103/PhysRevLett.122.166601 (2019).

[S6] Parkinson, P. *et al.* Transient Terahertz Conductivity of GaAs Nanowires. *Nano Lett.* **7,** 2162–2165; 10.1021/nl071162x (2007).
